# A fertility restorer gene, *Rf4*, widely used for hybrid rice breeding encodes a pentatricopeptide repeat protein

**DOI:** 10.1186/s12284-014-0028-z

**Published:** 2014-11-01

**Authors:** Tomohiko Kazama, Kinya Toriyama

**Affiliations:** Graduate School of Agricultural Science, Tohoku University, 1-1 Tsutsumidori-Amamiyamachi, Aoba-ku, 981-8555 Sendai Japan

**Keywords:** Cytoplasmic male sterility, Fertility restorer, Rice

## Abstract

**Background:**

Uncontrolled expression of a certain mitochondrial gene often causes cytoplasmic male sterility (CMS) in plants. This phenotype is prevented by the presence of a fertility restorer (*Rf*) gene in the nuclear genome. Such CMS/*Rf* systems have been successfully used for breedings of F_1_ hybrid cultivars. In rice, approximately 99% of F_1_ hybrid cultivars have been developed using a wild abortive type of CMS (WA-CMS) and its *Rf* genes. Recently, a newly identified mitochondrial gene, *orf352,* was reported as a WA-CMS-causing gene.

**Findings:**

We cloned and functionally characterized *Rf4*, a major *Rf* gene for WA-CMS. We revealed that *Rf4* encoded a pentatricopeptide repeat-containing protein and reduced the *orf352*-containing transcripts, thereby restoring pollen fertility.

**Conclusions:**

Through a map-based cloning, we have independently identified an allele of a recently reported *Rf4* gene and demonstrated that the fertility restoration is controlled sporophytically.

**Electronic supplementary material:**

The online version of this article (doi:10.1186/s12284-014-0028-z) contains supplementary material, which is available to authorized users.

## Findings

A wild abortive (WA)-type CMS has been almost exclusively used for breeding three-line hybrid rice and contributes to 10% of the total rice cultivated area worldwide (Li et al. [2007]; Barclay [2010]). Because of the great impact of WA-type CMS on agriculture, many studies have attempted to elucidate the CMS-causing gene in WA mitochondria and to determine the fertility restorer genes for WA-CMS. In 2013, a mitochondrial *orf352* (*WA352*) gene that confers WA-CMS was discovered; this gene encodes 352-amino-acids protein (Luo et al. [2013]). We also found a sequence variant of *orf352* in an RT102 CMS line derived from *O. rufipogon* (Okazaki et al. 2013). However, the *Rf* genes in nuclear genome have not yet been cloned, although two major *Rf* genes, *Rf3* and *Rf4,* have been mapped on the chromosomes 1 and 10, respectively (Lu et al. [1997]; Yao et al. [1997]; Tan et al. [2008]; Jing et al. [2001]; Ahmadikhah and Karlov [2006]; Ngangkham et al. [2010]; Suresh et al. [2012]). In this study, we report the cloning of *Rf4*. We also showed that the cloned *Rf4* reduced the *orf352*-containing transcripts and restored pollen fertility.

**Figure 1:**
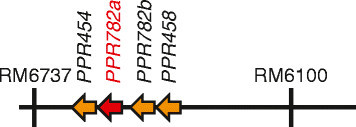
**Candidate genes present in the IR24 BAC clone.** Candidate genes and SSR markers in the IR24 BAC clone are indicated.

To identify the *Rf4* gene for WA-CMS, we performed map-based cloning of *Rf4* using a cultivar IR24 (Additional file 1: "Methods"), because this cultivar is known to be a strong restorer line for WA-CMS (Jing et al. [2001]). We delimited the *Rf4* candidate region between two SSR markers, SSR1045 and AT801, that corresponded to a 213-kb region of Nipponbare genome (Additional file 2: Table S1). While we were conducting the fine mapping, Ngangkham et al. ([2010]) reported that *Rf4* was located in a region between RM6737 and RM6100 with a distance of 104 kb in the Nipponbare genome, which further narrowed down the candidate region. We isolated a bacterial artificial chromosome (BAC) clones covering the corresponding region from IR24 genomic libraries (Figure 1) and determined their nucleotide sequences (Additional file 1: "Methods"). Because the presence of *Rf4* reduced the *orf352*-containing transcripts in the mitochondria, we identified the candidate gene according to the following two criteria, (i) it would encode a protein whose function is related to RNA metabolism, and (ii) it would encode a mitochondrial-targeting protein. The candidate genes that fulfilled the criteria were four new pentatricopeptide repeat (PPR)-encoding genes, *PPR454, PPR782a, PPR782b*, and *PPR458* (Figure 1). These genes were named on the number of amino acids that they encoded. PPR proteins are, in general, known to be involved in RNA regulation in the mitochondria and plastids (Schmitz-Linneweber and Small [2008]). We obtained genomic fragments containing *PPR454*, *PPR782a*, *PPR782b*, *or PPR458* from the IR24 BAC clone (Additional file 1: "Methods"; Additional file 3: Figure S1). We introduced each genomic fragment into a WA-CMS line, WAA, which carries a Taichung 65 nuclear background, and obtained at least twelve transgenic lines (Additional file 4: Figure S2).

**Figure 2:**
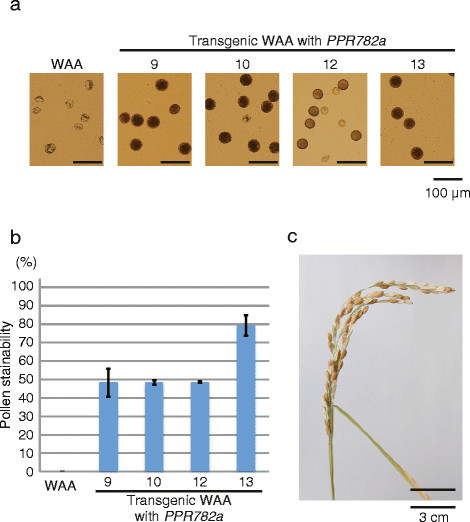
**Restoration of fertility in transgenic plants with** ***PPR782a*** **.** **(a)** Pollen grains stained with iodine-potassium iodide. **(b)** Pollen stainability of each transgenic plant. Error bars indicate the standard deviation. **(c)** Seed setting of the transgenic plant, No. 13.

The anthers of WA-CMS are milky white, slender, and stunted (Additional file 4: Figure S2) and contain shrunken pollen grains lacking starch accumulation ability, because of which they are not stained with 1% potassium iodide (Figure 2a). In contrast, anthers of fertile Taichung 65 are yellow and engorged and contain darkly stained pollen grains. In four of the thirteen transgenic plants with *PPR782a*, restorations of anther and pollen development and starch accumulation in pollen grains were observed (Figure 2a; Additional file 4: Figure S2). The percentage of stainable pollen grains (pollen stainability) was 48% for plant Nos. 9, 10, and 12, and 79% for plant No. 13 (Figure 2b). Especially, one of the plants (No. 13) showed 47% seed setting rate (Figure 2c). Restorations of anther and pollen development were not observed in the remaining nine plants. Introduction of three other genomic fragments, which contained *PPR782b*, *PPR454*, or *PPR458*, did not recover the anther and pollen morphology (Additional file 4: Figure S2), and resulted in 0% of pollen stainability and no seed setting. These data indicate that *Rf4* is the *PPR782a* gene.

T_1_ seeds were set by self-pollination. The progeny segregated into 8 fertile plants, all of which carried the introduced gene, and 7 male-sterile plants, all of which did not carry the introduced gene (Additional file 5: Figure S3). The appearance of null segregants indicated that *PPR782a* controlled the fertility restoration sporophytically. The pollen fertility was not completely restored by the introduced *Rf4* gene. Other fertility restorer genes, such as the *Rf3* gene on chromosome 1, might be necessary for fully restoration of WA-CMS (Suresh et al. [2012]).

The predicted amino acid sequence of PPR782A carries 18 repeats of PPR motif (Additional file 6: Figure S4) and is highly similar to RF1A for BT-CMS showing 86% identity (Kazama and Toriyama [2003]; Akagi et al. [2004]; Komori et al. [2004]; Kazama et al. [2008]). Amino acid identity between PPR782A and PPR782B was 94%, with a completely identical region in the N- and C-terminal ends. A non-restorer line, Nipponbare, contained a putative allele of *PPR782a*, which is encoded by the Rice Annotation Project (RAP) locus ID Os10g0495200. The amino acid sequence of PPR782A_Nipponbare shows 95% identity to that of PPR782A_IR24 (Additional file 6: Figure S4). Some amino acid substitutions might be crucial for the function of the PPR protein, as was reported for other PPR-type *Rf* genes in petunia (Bentolila et al. [2002]) and radish (Brown et al. [2003]; Desloire et al. [2003]; Koizuka et al. [2003]).

**Figure 3:**
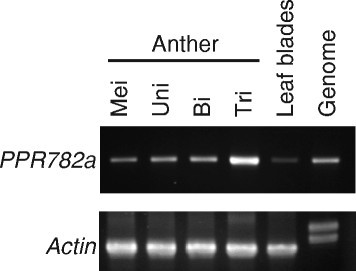
***PPR782a*** **is expressed in various tissues with the highest expression in mature anthers.** RT-PCR analysis of *PPR782a* in anthers at the meiotic stage (Mei), uni-nucleate microspore stage (Uni), bi-cellular pollen stage (Bi), and tri-cellular pollen stage (Tri), and in the leaf blades. PCR results obtained using genomic DNA is also shown.

Expression of *Rf4* was assessed using RT-PCR and gene-specific primers (Additional file 1: "Methods"). *Rf4* was expressed weakly in leaf blades. Expression was increased in the course of anther development, with the highest levels noted in the anthers at the tri-cellular pollen stage (Figure 3). *Rf4* has been proposed to express highly in the tapetum, because the fertility restoration was sporophytically controlled. Our results of expression analysis indicated that *Rf4* might play a gametophytic role in the maturation of pollen grains after tapetum degradation, as well as a sporophytic role.

**Figure 4:**
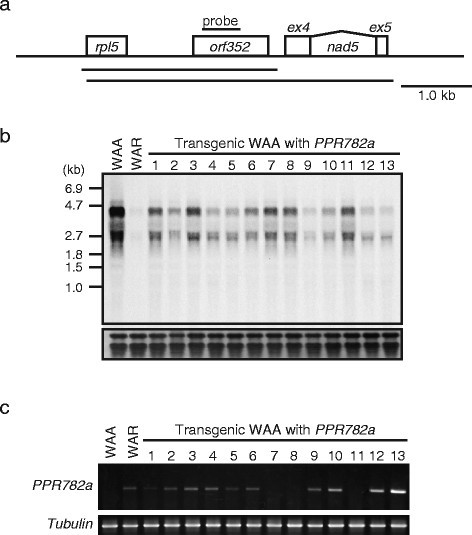
**Reduction of** ***orf352*** **-containing RNA in transgenic plants with** ***PPR782a*** **. (a)** Genomic structure around *orf352* and *orf352*-containing transcripts. *orf352* is located between *rpl5* and *nad5* _*exon4* _*exon5. orf* 352 is transcribed as both 4.7-kb and 2.5-kb RNA. Horizontal lines represent both transcripts containing *orf352*. **(b)** Northern blot analysis of RNA isolated from leaf blades of a cytoplasmic male sterility (CMS) line (WAA), a fertility restorer line (WAR), and transgenic WAA plants with *PPR782a*. Staining of ribosomal RNA is shown as a loading control. **(c)** Expression of the introduced *PPR782a* and *tubulin* detected by RT-PCR.

In a recent report on the WA-CMS-associated mitochondrial gene, *orf352* (*WA352*), RF4 was proposed to function post-transcriptionally, leading to the degradation of *orf352*-containing transcripts, half of which were co-transcribed with the upstream gene *rpl5* encoding the ribosomal protein large subunit 5 (*rpl5*-*orf352* transcripts; Figure 4a), whereas RF3 acted post-translationally and suppressed translation of *orf352* (Luo et al. [2013]). To determine whether the cloned *Rf4* reduced *rpl5*-*orf352* transcripts, we performed northern blot analysis by using RNA extracted from the leaf blades (Additional file 1: "Methods"). In a WA-CMS plant, WAA, we detected two strong signals of 4.5- and 2.7-kb bands corresponding to the *rpl5*-*orf352* and *orf352* transcripts, respectively (Figure 4b), as reported (Luo et al. [2013]). In a fertility restorer line, WAR, the signals of both the bands almost disappeared (Figure 4b). In four transgenic plants (Nos. 9, 10, 12, and 13) showing recovery of anther morphology and pollen stainability, the amount of the two transcripts decreased (lanes 9, 10, 12, and 13; Figure 4b). These four plants showed higher expression of the introduced *PPR782a* gene as detected by RT-PCR (Figure 4c). Northern blot analyses performed using *cox1* and *atp9* probes revealed that the introduction of *PPR782a* did not affect the accumulation of these transcripts (Additional file 7: Figure S5). These results suggested that *Rf4* was involved in the reduction of *orf352*-containing transcripts.

RF4 is considered to recognize and bind *orf352*-containing transcripts and promote the degradation of *orf352*-containing transcripts to avoid ORF352-mediated premature programmed cell death and consequent male sterility (Luo et al. [2013]). Finding a nuclear *Rf* factor provide novel insights into reconciliation between mitochondria and nuclei in agronomically important crops, and has practical implications for production of hybrid rice.

During preparation of this manuscript, Tang et al. ([2014]) have reported a short letter entitled as "The rice restorer *Rf4* for wild-abortive cytoplasmic male sterility encodes a mitochondrial-localized PPR protein that functions in reduction of *WA352* transcripts". Their study turned to be essentially the same to ours. They used a PCR-amplified genomic clone of cv. Minghui 63 for a complementation test, but did not investigate the self-pollinated progeny. In contrast, our study used the genomic clones isolated from BAC libraries of IR24 and examined the segregation in the T_1_ plants, showing that the fertility restoration was controlled sporophytically (Additional file 5: Figure S3). The nucleotide sequence of the *RF4* allele of IR24 in our study is completely identical to that presented in Supplementary Figure of Tang et al. ([2014]). An adjacent gene, *PPR782b*, identified in our study has not been reported in their study. Thus our study is not just a confirmation of Tang et al. ([2014]), but further provides more information.

### Accession codes

The nucleotide sequences of *PPR454*, *PPR782a*, *PPR782b*, and *PPR458* have been deposited at the DDBJ under accession numbers [AB900791 to AB900794].

## Authors' contributions

TK performed the experiments and drafted the manuscript. KT designed and supervised the study and revised the manuscript. Both authors read and approved the final draft of the manuscript.

## Additional files

## Electronic supplementary material

Additional file 1:**Methods.** Table S2. Primer Sequences used for RT-PCR and probe synthesis for northern blot analysis. (DOCX 90 KB)

Additional file 2: Table S1.: Mapping of *Rf4*. Primer information used for mapping *Rf4* and number of plants in the range of percentage of seed setting in F_2_ plants homozygously carrying Taichung 65 allele at the designated SSR markers. (DOCX 92 KB)

Additional file 3: Figure S1.: Candidate genes (*PPR454*, *PPR782a*, *PPR782b* and *PPR458*) and the genomic clones used for the complementation test. The nucleotide sequences are deposited at the DDBJ under accession numbers AB900791, AB900792, AB900793, and AB900794, respectively. (PDF 260 KB)

Additional file 4: Figure S2.: Restoration of anther morphology in transgenic plants with *PPR782a*. Anthers of WAA are milky white, slender, and stunted, whereas those of T65 are yellow and engorged. *Number of plants with recovered anther development/number of total transgenic plants is indicated in parenthesis. (PDF 3 MB)

Additional file 5: Figure S3.: Segregation of fertile and sterile plants in T_1_ progeny. (a) Segregation of the transgenes (*PPR782a* and *HPT*) in each T_1_ plant obtained by self-pollination of the No. 13 plant. The lowest panel indicates control PCR amplifying *tubulin* genetic region. Seed setting rates of each T_1_ plant are shown under the panel. (b) Representative anthers of T_1_ plants with the introduced *PPR782a* were yellow and engorged. On the other hand, null segregants produced stunted anthers as those of WAA. (PDF 1 MB)

Additional file 6: Figure S4.: Amino acid sequence of PPR782A_IR24 aligned with PPR782B_IR24 and Os10g0495200 of Nipponbare. Alignments were performed using ClustalW2.1. The eighteen PPR motifs are included in gray boxes. A mitochondrial targeting signal peptide predicted by MitoProt II is shown in red. (PDF 284 KB)

Additional file 7: Figure S5.: Northern blot analysis of *cox1* and *atp9*. RNA was isolated from leaf blades of a CMS line (WAA), a fertility restorer line (WAR), and transgenic WAA plants with *PPR782a*. Staining of ribosomal RNA is shown as a loading control. (PDF 2 MB)

Below are the links to the authors’ original submitted files for images.

Authors’ original file for figure 1

Authors’ original file for figure 2

Authors’ original file for figure 3

Authors’ original file for figure 4

## Abbreviations

BAC: Bacterial artificial chromosome
BT: Boro-Taichung
CMS: Cytoplasmic male sterility
PPR: Pentatricopeptide repeat
RT-PCR: Reverse-transcriptase-polymerase chain reaction
SSR: Simple sequence repeat
WA: Wild abortive
