# Supplementary material for: A fertility restorer gene, Rf4, widely used for hybrid rice breeding encodes a pentatricopeptide repeat protein
Source: Rice (N Y). 2014 Nov 1;7:28. doi: 10.1186/s12284-014-0028-z (PMC4884050; doi:10.1186/s12284-014-0028-z)
Supplement: Supplementary file 1 — Additional file 1:Methods. Table S2. Primer Sequences used for RT-PCR and probe synthesis for northern blot analysis. (DOCX 90 KB) [file 12284_2014_28_MOESM1_ESM.docx]

**Methods**

**Plant materials and mapping of *Rf4***

A WA-CMS line, WAA, has the nuclear background of a *japonica* cultivar Taichung 65(Fujii et al. 2010). A fertility restorer line, WAR, was obtained by crossing a WA-CMS line and an Asominori/IR24 recombinant inbred line, RIL-37, in which the *Rf4* region of chromosome 10 was derived from IR24, and the *Rf3* region on chromosome 1 was from Asominori (Kubo et al. 1999). Mapping population consisting of 331 F_2_ plants was obtained by crossing WAR and Taichung 65. Genomic DNA extraction and PCR analysis were performed as previously described (Kazama and Toriyama 2003). A fertile plant carrying homozygous Taichung 65 alleles of SSR markers (Additional file 2: Table S1) on one side of a candidate region were regarded as a recombinant plant.

**Complementation test**

Construction and screening of the BAC library of IR24 were performed at the National Institute of Agrobiological Sciences (Tsukuba, Japan). The sequences of the BAC clones covering the candidate region were determined using a GS-FLX system (Roche) at TAKARA BIO INC (Ohtsu, Japan).

The BAC clone was digested with *Eco* RI, *Kpn* I, or *Xba* I, and each fragment containing each gene was identified and inserted into pBlueScript II vector. After confirming the nucleotide sequences using the CEQ8000 genetic analysis system (Beckman Coulter), each fragment was transferred into the binary vector pZH2B (provided by Dr. Masaharu Kuroda, National Agricultural Research Center, Joetsu, Japan) (Kuroda et al. 2010). The resulting each vector was introduced into a WAA line by the method of *Agrobacterium*-mediated transformation (Toki et al. 2006). Spikelets collected a day before anthesis were harvested, and pollen grains were stained with a 1% (w/v) iodine-potassium iodide solution to observe starch accumulation (Itabashi et al. 2011).

**Expression analysis**

Total RNA was isolated from the anthers at the meiotic, uni-nucleate microspore and bi-cellular and tri-cellular pollen stages and from the young leaves and calli by using RNeasy Plant Mini Kit (Qiagen, Tokyo, Japan), as described previously (Itabashi et al. 2011). DNA was eliminated using RNase-free DNase I (TAKARA BIO INC., Ohtsu, Japan). Primers used for the detection of each transcript are listed in TableS2. PCR conditions used were as follows: 94°C for 1 min, followed by 30 cycles of 94°C for 30 s, 57°C for 30 s, 72°C for 30 s, and final extension at 72°C for 3 min. The primer set for *actin* is listed in Additional file 8: Table S2.

For northern blot analysis, *orf352*, *cox1* and *atp9*, each probe was synthesized using primers listed in Table S2. Total RNA was isolated from young leaf blades. Northern blot analysis was performed using 10 µg of each RNA as previously described (Kazama and Toriyama 2003, Kazama et al. 2008).

Fujii S, Yamada M, Fujita M, Itabashi E, Hamada K, Yano K, Kurata N, Toriyama K (2010) Cytoplasmic-nuclear genomic barriers in rice pollen development revealed by comparison of global gene expression profiles among five independent cytoplasmic male sterile lines. Plant Cell Physiol 51: 610-620. doi:10.1093/pcp/pcq026

Itabashi E, Iwata N, Fujii S, Kazama T, Toriyama K (2011) The fertility restorer gene, *Rf2*, for Lead Rice-type cytoplasmic male sterility of rice encodes a mitochondrial glycine-rich protein. Plant J 65: 359-367. doi:10.1111/J.1365-313x.2010.04427.X

Kubo T, Nakamura K, Yoshimura A (1999) Development of a series of *Indica* chromosome segment substitution lines in *Japonica* background of rice. Rice Genet Newslett 16: 104-106.

Kuroda M, Kimizu M, Mikami C (2010) A simple set of plasmids for the production of transgenic plants. Biosci Biotech Biochem 74: 2348-2351. doi:10.1271/Bbb.100465

Ngangkham U, Parida SK, De S, Kumar KAR, Singh AK, Singh NK, Mohapatra T (2010) Genic markers for wild abortive (WA) cytoplasm based male sterility and its fertility restoration in rice. Mol Breed 26: 275-292. doi:10.1007/S11032-010-9397-1

Toki S, Hara N, Ono K, Onodera H, Tagiri A, Oka S, Tanaka H (2006) Early infection of scutellum tissue with *Agrobacterium* allows high-speed transformation of rice. Plant J 47: 969-976. doi:10.1111/J.1365-313x.2006.02836.X

**Table S2**

Primer sequences used for RT-PCR and probe synthesis for northern blot analysis.

| Target gene | Forward primer | Reverse primer |
| --- | --- | --- |
| *PPR782a* | ACATGATGGCTGATGATCGA | CTTCATCCATCTTACCACCT |
| *OsActin* | AACTGGGATGATATGGAGAA | CCTCCAATCCAGACACTGTA |
| *orf352* | ATGACGAGAGATAGAATGAG | GCCGTACGGGGGTTGTCCAA |
| *cox1* | TGTACTTTCTATGGGAGCCG | TTGATAGCTGGAAGTTCTCC |
| *atp9* | TGAATTGAGAAAGCCGGTGG | TCAGAAAGGCCATCATTGGG |
| *Tubulin* | TGGTCGGATTCGCCCCGCTG | TTACATGTCGTCAGCCTCCT |
