# Supplementary material for: A fertility restorer gene, Rf4, widely used for hybrid rice breeding encodes a pentatricopeptide repeat protein
Source: Rice (N Y). 2014 Nov 1;7:28. doi: 10.1186/s12284-014-0028-z (PMC4884050; doi:10.1186/s12284-014-0028-z)
Supplement: Supplementary file 2 — Additional file 2: Table S1.: Mapping of Rf4. Primer information used for mapping Rf4 and number of plants in the range of percentage of seed setting in F2 plants homozygously carrying Taichung 65 allele at the designated SSR markers. (DOCX 92 KB) [file 12284_2014_28_MOESM2_ESM.docx]

**Table S1** Mapping of *Rf4*

Primer information used for mapping *Rf4* and number of plants in the range of percentage of seed setting in F_2_ plants homozygously carrying Taichung 65 allele at the designated SSR markers.

| Marker | Position in IRGSP build5* | Forward/Reverse  primer sequence | Number of plants | | | |
| --- | --- | --- | --- | --- | --- | --- |
|  |  |  | 0%  –  10% | 10%  –  30% | 30%  –  70% | 70%  –  100% |
| SSR1041 | 18,503 | GTAGGGACCAGGAGATGG /  CAGGATCACCGTCGTCGT | 34 | 0 | 1 | 0 |
| AT122 | 19,153 | ATAGAGCGAAGATTTAGCGC /  CTAAGAGGCTCTACCATGCA | 32 | 1 | 0 | 0 |
| AT702 | 19,224 | AACCGGCACATATAAGACCG /  GCAGGACATGGGAGATATCA | 32 | 1 | 0 | 0 |
| SSR1045 | 19,243 | AGCTCAACTCGCAACTCCC /  CCATCTCCTCTTTCACCTCG | 33 | 1 | 0 | 0 |
| RM6737 | 19,281 | Refer to Ahmadikhah et al. (2006) and Ngangkham et al. (2010) | ND | | | |
| SSR1049 | 19,385 | TCCTCTACCAGTACCGCACC /  GCTGGATCACAGATCATTGC | 32 | 0 | 0 | 0 |
| RM6100 | 19,385 | Refer to reference Ngangkham et al. (2010) | ND | | | |
| RF1 locus | 19,449 |  |  |  |  |  |
| AT801 | 19,456 | TGCCGGTATTTTGGTACGTC /  TGGATTCCTAATTGCAGCTC | 39 | 4 | 3 | 6 |
| AB443 | 19,456 | Refer to Ahmadikhah et al. (2006) | ND | | | |

*Positions of SSR markers based on International Rice Sequencing Project (IRGSP) build5 pseudomolecules.
