# Supplementary material for: A fertility restorer gene, Rf4, widely used for hybrid rice breeding encodes a pentatricopeptide repeat protein
Source: Rice (N Y). 2014 Nov 1;7:28. doi: 10.1186/s12284-014-0028-z (PMC4884050; doi:10.1186/s12284-014-0028-z)
Supplement: Supplementary file 3 — Additional file 3: Figure S1.: Candidate genes (PPR454, PPR782a, PPR782b and PPR458) and the genomic clones used for the complementation test. The nucleotide sequences are deposited at the DDBJ under accession numbers AB900791, AB900792, AB900793, and AB900794, respectively. (PDF 260 KB) [file 12284_2014_28_MOESM3_ESM.pdf]

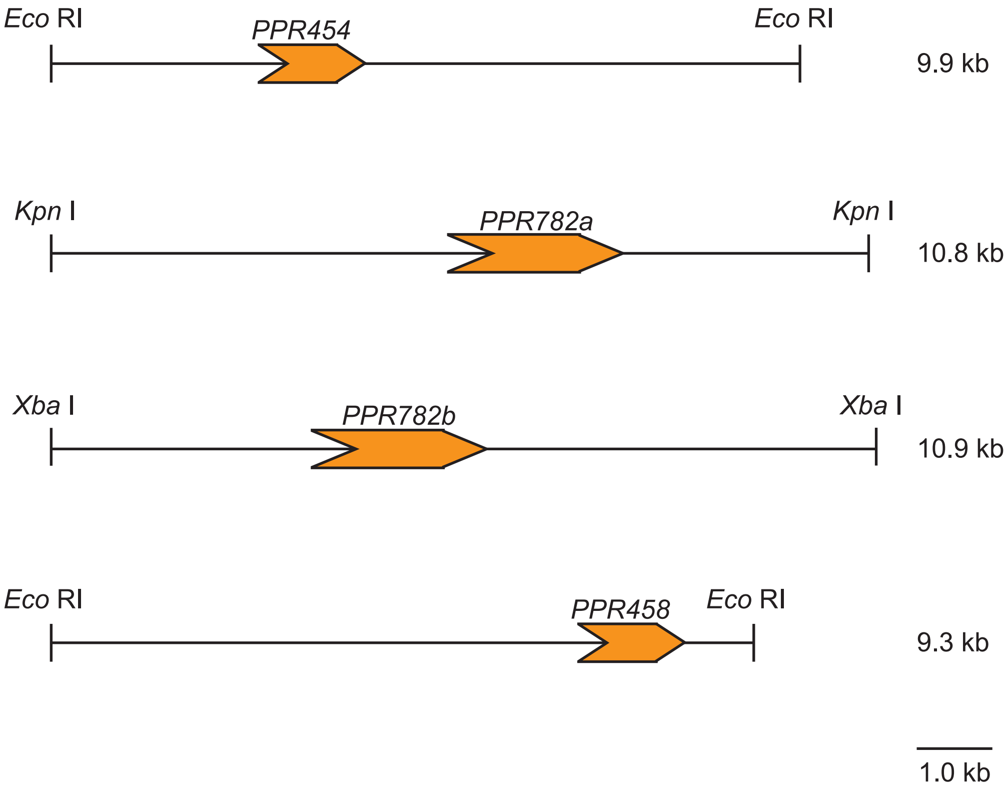

Figure S1
Candidate genes (*PPR454*, *PPR782a*, *PPR782b* and *PPR458*) and the genomic clones used for the complementation test.

The nucleotide sequences are deposited at the DDBJ under accession numbers AB900791, AB900792, AB900793, and AB900794, respectively.
