# Supplementary material for: A fertility restorer gene, Rf4, widely used for hybrid rice breeding encodes a pentatricopeptide repeat protein
Source: Rice (N Y). 2014 Nov 1;7:28. doi: 10.1186/s12284-014-0028-z (PMC4884050; doi:10.1186/s12284-014-0028-z)
Supplement: Supplementary file 4 — Additional file 4: Figure S2.: Restoration of anther morphology in transgenic plants with PPR782a. Anthers of WAA are milky white, slender, and stunted, whereas those of T65 are yellow and engorged. *Number of plants with recovered anther development/number of total transgenic plants is indicated in parenthesis. (PDF 3 MB) [file 12284_2014_28_MOESM4_ESM.pdf]

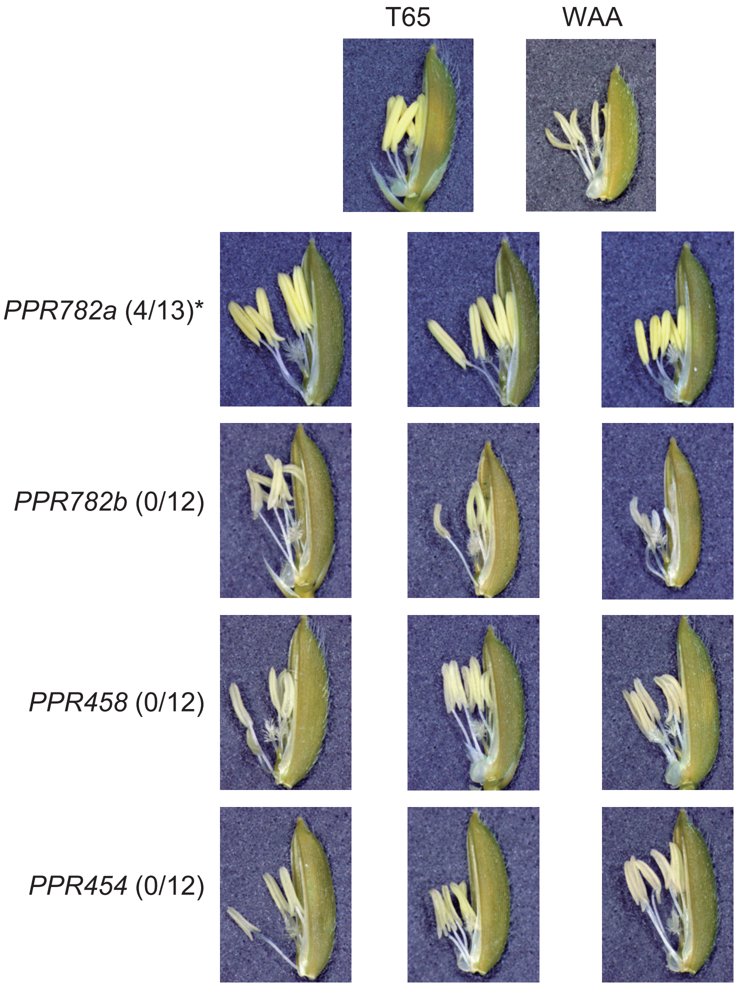

Figure S2
Restoration of anther morphology in transgenic plants with *PPR782a*.

Anthers of WAA are milky white, slender, and stunted, whereas those of T65 are yellow and engorged. *Number of plants with recovered anther development/number of total transgenic plants is indicated in parenthesis. Four out of 13 transgenic plants with *PPR782a* produced yellow and engorged anthers, whereas all the transgenic plants with *PPR782b*, *PPR458*, and *PPR454* produced stunted anthers as those of WAA.
