# Supplementary material for: A fertility restorer gene, Rf4, widely used for hybrid rice breeding encodes a pentatricopeptide repeat protein
Source: Rice (N Y). 2014 Nov 1;7:28. doi: 10.1186/s12284-014-0028-z (PMC4884050; doi:10.1186/s12284-014-0028-z)
Supplement: Supplementary file 5 — Additional file 5: Figure S3.: Segregation of fertile and sterile plants in T1 progeny. (a) Segregation of the transgenes (PPR782a and HPT) in each T1 plant obtained by self-pollination of the No. 13 plant. The lowest panel indicates control PCR amplifying tubulin genetic region. Seed setting rates of each T1 plant are shown under the panel. (b) Representative anthers of T1 plants with the introduced PPR782a were yellow and engorged. On the other hand, null segregants produced stunted anthers as those of WAA. (PDF 1 MB) [file 12284_2014_28_MOESM5_ESM.pdf]

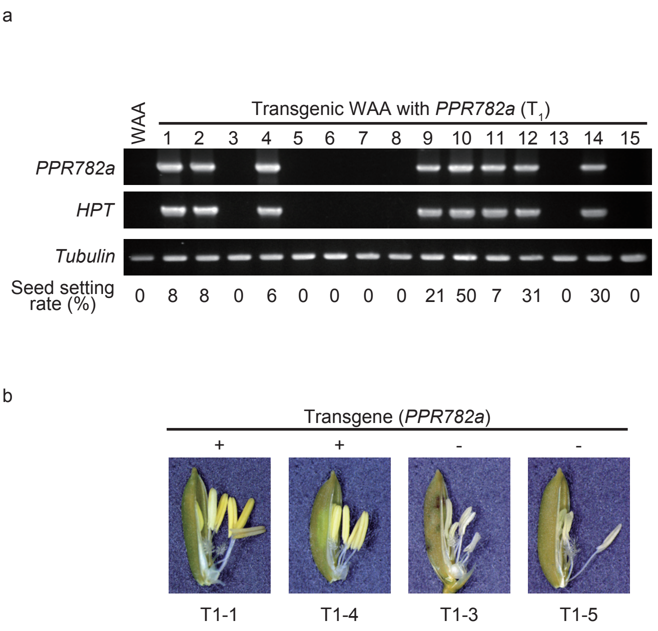

Figure S3

Segregation of fertile and sterile plants in T_1 progeny.

(a) Segregation of the transgenes (*PPR782a* and *HPT*) in each T_1 plant obtained by self-pollination of No. 13 plant. The lowest panel indicates control PCR amplifyng *tubulin* genetic region. Seed setting rates of each T_1 plant are shown under the panel. (b) Representative anthers of T_1 plants with the introduced *PPR782a* are yellow and engorged. On the other hand, null segregants produced stunted anthers as those of WAA.
