# Supplementary material for: A fertility restorer gene, Rf4, widely used for hybrid rice breeding encodes a pentatricopeptide repeat protein
Source: Rice (N Y). 2014 Nov 1;7:28. doi: 10.1186/s12284-014-0028-z (PMC4884050; doi:10.1186/s12284-014-0028-z)
Supplement: Supplementary file 6 — Additional file 6: Figure S4.: Amino acid sequence of PPR782A_IR24 aligned with PPR782B_IR24 and Os10g0495200 of Nipponbare. Alignments were performed using ClustalW2.1. The eighteen PPR motifs are included in gray boxes. A mitochondrial targeting signal peptide predicted by MitoProt II is shown in red. (PDF 284 KB) [file 12284_2014_28_MOESM6_ESM.pdf]

```
PPR782A_IR24   MARRVPTRPR GGGGGGVPRS EGSIQGRGGR AGGSGAEDAR HVFDELLRRG RGASIYGLNR ALADVARHSP AAAVSRYNRM ARAGAGKVTP
PPR782B_IR24   MARRVPTRPR GGGGGGVPRS EGSIQGRGGR AGGSGAEDAR HVFDELLRRG RGASIYGLNR ALADVARHSP AAAVSRYNRM ARAGADEVTP
Os10g0495200   MARRVPTRPR GGGGGGVPRS EGSIQGRGGR AGGSGAEDAR HVFDELLRRG RGASIYGLNR ALADVARHSP AAAVSRYNRM ARAGAGKVTP
               ********** ********** ********** ********** ********** ********** ********** ********** *****  ***

PPR782A_IR24   HTYAILIGCCCRAGRLDLGFAALGNVVKKGFRVDA
PPR782B_IR24   CTYGILIGCCCRAGRLDLGFAALGNVIKKGFRVEA
Os10g0495200   HTYAILIGCCCRAGRLDLGFAALGNVVKKGFRVDA
                ** ********************** ****** *

PPR782A_IR24   ITFTPLLKGLCADKRTSDAMDIVLRRMTELGCIPD V
PPR782B_IR24   ITFTPLLKGLCADKRTSDAMDIVLRRMTELGCIPN V
Os10g0495200   ITFTPLLKGLCADKRTSDAMDIVLRRMTELGCIPD V
               **********************************  *

PPR782A_IR24   FSYNILLKGLCDENRSQEALELLHMMADDRGGGSP PDV
PPR782B_IR24   FSCTILLKGLCDENGSQEALELLHMMADDRGGGSP PDV
Os10g0495200   FSYNNLLKGLCDENRSQEALELLHMMADDRGGGSP PDV
               **   ********* ******************** ***

PPR782A_IR24   VSYNTVLNGFFKEGDSDKAYSTYHEMLDRGILPDV
PPR782B_IR24   VSYTTVINGFFKEGDLDKAYSTYHEMLDRGISPNV
Os10g0495200   VSYNTVLNGFFKEGDSDKAYSTYHEMLDRGILPDV
               *** ** ******** *************** * *

PPR782A_IR24   VTYSSIIAALCKAQAMDKAMEVLTTMVKNGVMPDC
PPR782B_IR24   VTYSSIIAALCKAQAMDKAMEVLNTMVKNGVMPDC
Os10g0495200   VTYSSIIAALCKAQAMDKAMEVLNTMVKNGVMPDC
               *********************** ***********

PPR782A_IR24   MTYTSIMHGYCSSGQPKEAIGFLKKMRSDGVKPNV
PPR782B_IR24   MTYNSILHGYCSSGQPKEAIGTLKKMRSDGVEPNV
Os10g0495200   MTYNSILHGYCSSGQPKEAIGTLKKMRSDGVEPNV
               *** ** ************** ********* ***

PPR782A_IR24   FTYRSLMNYLCKNGRSTEARKIFDSMTKRGLEPDI
PPR782B_IR24   FTYRSLMNYLCKNGRSTEARKIFDSMTKRGLEPDI
Os10g0495200   VTYSSLMNYLCKNGRSTEARKIFDSMTKRGLEPDI
                ** *******************************

PPR782A_IR24   ATYRTLLQGYATKGALVEMHALLDLMVRNGIQPDH
PPR782B_IR24   ATYGTLLQGYATKGALVEMHALLDLMVRNGIQPDH
Os10g0495200   ATYRTLLQGYATKGALVEMHALLDLMVRNGIQPDH
               *** *******************************

PPR782A_IR24   HVFNILICAYAKQEKVDQAMLVFSKMRQHGLNPNV
PPR782B_IR24   HVFNILICAYAKQEKVDQAMLVFSKMRQHGLNPNV
Os10g0495200   HVFNILICAYAKQEKVDQAMLVFSKMRQHGLNPNV
               ***********************************

PPR782A_IR24   VTYGTVIDVLCKSGSVDDAMLYFEQMIDEGLTPNI
PPR782B_IR24   VTYGTVIDVLCKSGSVDDAMLYFEQMIDEGLTPNI
Os10g0495200   VCYGTVIDVLCKSGSVDDAMLYFEQMIDEGLTPNI
               * *********************************

PPR782A_IR24   IVYTSLIHGLCTYDKWEKAEELFFKMLDSGICPNT
PPR782B_IR24   IVYTSLIHGLCTYDKWEKAEELFFKMLDSGICLNT
Os10g0495200   IVYTSLIHGLCTCDKWDKAEELILEMLDRGICLNT
               ************ *** *****   *** *** **

PPR782A_IR24   VFFSSIISNLCKEGRVIESEKLFDLMVRIGVKPNV
PPR782B_IR24   VFFSSIISNLCKEGRVIESEKLFDLMVRIGVKPNV
Os10g0495200   IFFNSIIDSHCKEGRVIESEKLFDLMVRIGVKPDI
                ** ***   ***********************

PPR782A_IR24   ITYNTLIDGCCLGGKMDEAMKLLSGMVSVGLKPNT
PPR782B_IR24   ITYNTLIDGCCLAGKMDEATKLLASMVSVGVKPDI
Os10g0495200   ITYNTLIDGCCLAGKMDEATKLLASMVSVGVKPDI
               ************ ****** ***  ***** **

PPR782A_IR24   VTYSTLINGYCKISRMEDALVLFKEMESSGVSPDI
PPR782B_IR24   VTYGTLINGYCRVSRMDDALALFKEMVSSGVSPNI
Os10g0495200   VTYGTLINGYCRVSRMDDALALFKEMVSSGVSPNI
               *** *******  *** *** ***** ****** *

PPR782A_IR24   ITYNIILQGLFQTRRTAAAKELYVSITKSGTQLEL
PPR782B_IR24   ITYNIILQGLFHTRRTAAAKELYVSITKSGTQLEL
Os10g0495200   ITYNIILQGLFHTRRTAAAKELYVSITKSGTQLEL
               *********** ***********************

PPR782A_IR24   STYNIILHGLCKNNLTDEALRMFQNLCLTDLQLET
PPR782B_IR24   STYNIILHGLCKNNLTDEALRMFQNLCLTDLQLET
Os10g0495200   STYNIILHGLCKNNLTDEALRMFQNLCLTDLQLET
               ***********************************

PPR782A_IR24   RTFNIMIGALLKCGRMDEAKDLFAAHSANGLVPDV
PPR782B_IR24   RTFNIMIGALLKCGRMDEAKDLFAAHLANGLVPDV
Os10g0495200   RTFNIMIGALLKCGRMDEAKDLFAAHSANGLVPDV
               ************************** ********

PPR782A_IR24   WTYSLMAENLIEQGSLEELDDLFLSMEENGCSADS
PPR782B_IR24   WTYSLMAENLIEQGSLEELDDLFLSMEENGCSADS
Os10g0495200   RTYSLMAENLIEQGSLEELDDLFLSMEENGCSADS
                **********************************

PPR782A_IR24   RMLNSIVRKL LQRGDITRAG TYLFMIDEKH FSLEASTASF LLESSPIVWE QISRIS
PPR782B_IR24   RMLNSIVRKL LQRGDITRAG TYLFMIDEKH FSLEASTASF LLESSPIVWE QISRIS
Os10g0495200   RMLNSIVRKL LQRGDITRAG TYLFMIDEKH FSLEASTASF LLESSPIVWE QISRIS
               ********** ********** ********** ********** ********** ******
```

Figure S4

Amino acid sequence of PPR782A_IR24 aligned with PPR782B_IR24 and Os10g0495200 of Nipponbare.

Alignments were performed using ClustalW2.1. The eighteen PPR motifs are included in gray boxes.
A mitochondrial targeting signal peptide predicted by MitoProt II is shown in red.
