# Supplementary material for: A fertility restorer gene, Rf4, widely used for hybrid rice breeding encodes a pentatricopeptide repeat protein
Source: Rice (N Y). 2014 Nov 1;7:28. doi: 10.1186/s12284-014-0028-z (PMC4884050; doi:10.1186/s12284-014-0028-z)
Supplement: Supplementary file 7 — Additional file 7: Figure S5.: Northern blot analysis of cox1 and atp9. RNA was isolated from leaf blades of a CMS line (WAA), a fertility restorer line (WAR), and transgenic WAA plants with PPR782a. Staining of ribosomal RNA is shown as a loading control. (PDF 2 MB) [file 12284_2014_28_MOESM7_ESM.pdf]

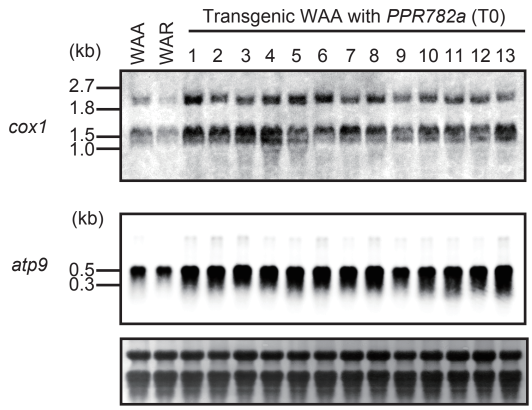

Figure S5
Northern blot analysis of *cox1* and *atp9*.

RNA was isolated from leaf blades of a CMS line (WAA), a fertility restorer line (WAR), and transgenic WAA plants with *PPR782a*. Staining of ribosomal RNA is shown as a loading control.
