# Supplementary figures and images for: A fertility restorer gene, Rf4, widely used for hybrid rice breeding encodes a pentatricopeptide repeat protein
Source: Rice (N Y). 2014 Nov 1;7:28. doi: 10.1186/s12284-014-0028-z (PMC4884050; doi:10.1186/s12284-014-0028-z)

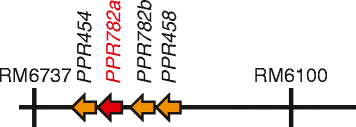

Supplement: Supplementary file 8 — Authors’ original file for figure 1 [file 12284_2014_28_MOESM8_ESM.gif]

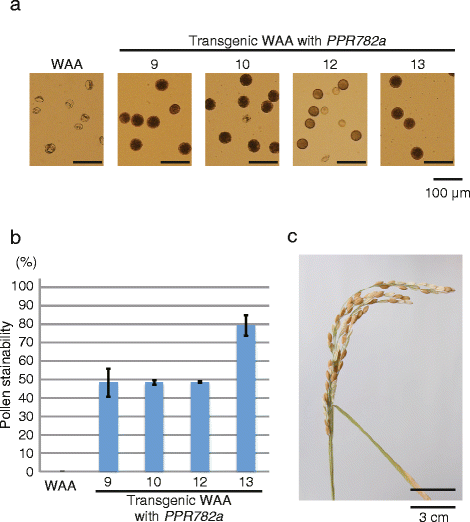

Supplement: Supplementary file 9 — Authors’ original file for figure 2 [file 12284_2014_28_MOESM9_ESM.gif]

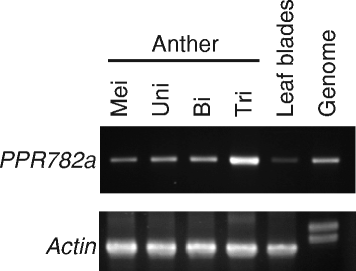

Supplement: Supplementary file 10 — Authors’ original file for figure 3 [file 12284_2014_28_MOESM10_ESM.gif]

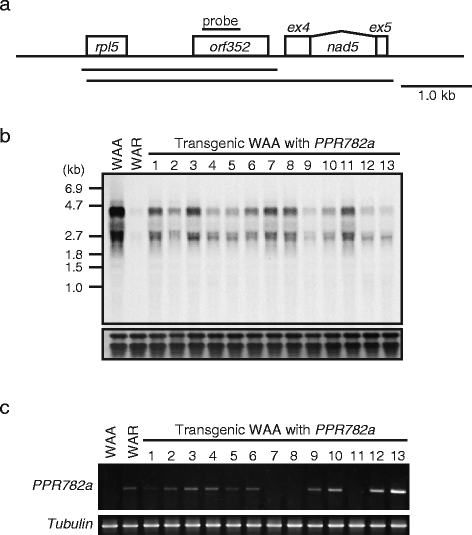

Supplement: Supplementary file 11 — Authors’ original file for figure 4 [file 12284_2014_28_MOESM11_ESM.gif]
